# Generating an organ-deficient animal model using a multi-targeted CRISPR-Cas9 system

**DOI:** 10.1038/s41598-024-61167-3

**Published:** 2024-05-09

**Authors:** Jonathan Jun-Yong Lim, Yamato Murata, Shunsuke Yuri, Kohei Kitamuro, Taro Kawai, Ayako Isotani

**Affiliations:** 1 Laboratory of Organ Developmental Engineering, Division of Biological Science, Graduate School of Science and Technology, Nara Institute of Science and Technology, 8916-5 Takayama-cho, Ikoma, Nara 630-0912 Japan; 2 Laboratory of Molecular Immunobiology, Division of Biological Science, Graduate School of Science and Technology, Nara Institute of Science and Technology, 8916-5 Takayama-cho, Ikoma, Nara 630-0912 Japan; 3 Department of Physiology, Yong Loo Lin School of Medicine, National University of Singapore, Singapore City, Singapore; 4 Life Science Collaboration Center (LiSCo), Nara Institute of Science and Technology, 8916-5 Takayama-cho, Ikoma, Nara 630-0192 Japan

**Keywords:** Biological techniques, Cell biology, Developmental biology, Stem cells

## Abstract

Gene-knockout animal models with organ-deficient phenotypes used for blastocyst complementation are generally not viable. Animals need to be maintained as heterozygous mutants, and homozygous mutant embryos yield only one-fourth of all embryos. In this study, we generated organ-deficient embryos using the CRISPR-Cas9-sgRNA^ms^ system that induces cell death with a single-guide RNA (sgRNA^ms^) targeting multiple sites in the genome. The Cas9-sgRNA^ms^ system interrupted cell proliferation and induced cell ablation in vitro. The mouse model had Cas9 driven by the *Foxn1* promoter with a ubiquitous expression cassette of sgRNA^ms^ at the *Rosa26* locus (*Foxn1*^*Cas9*^; *Rosa26_ms*). It showed an athymic phenotype similar to that of nude mice but was not hairless. Eventually, a rat cell-derived thymus in an interspecies chimera was generated by blastocyst complementation of *Foxn1*^*Cas9*^; *Rosa26_ms* mouse embryos with rat embryonic stem cells. Theoretically, a half of the total embryos has the Cas9-sgRNA^ms^ system because Rosa26_ms could be maintained as homozygous.

## Introduction

With increase in the demand for organ transplants in recent decades, organ shortage due to insufficient number of organ donors has become a worldwide problem. This deficit may be compensated by growing transplantable human organs in laboratory animals using the blastocyst complementation method. Injecting pluripotent stem cells (PSCs) into organ-deficient embryos can regenerate fully developed organs^1–5^. Organ-deficient embryos are hence necessary for blastocyst complementation.

Current methods for generating organ-deficient embryos include knockout (KO) of organ-specific genes^1–8^ and conditional cell ablation with diphtheria toxin A (DTA) using the Cre/LoxP system^9–12^. However, most organ-deficient animal models have a lethal phenotype. Therefore, animals need to be maintained as heterozygous mutants or in two or more lines, and organ-deficient embryos yield only a quarter or less of the total number of embryos in most crossing combinations. Thus, using gene-KO and DTA-based cell ablation models for blastocyst complementation is labor-efficient and costly. Most genes that control organ and tissue development are also involved in other processes. The injection of PSCs rescued KO cells in some gene-KO models, resulting in organs containing a mixture of host- and PSC-derived tissues.

**Figure 1:**
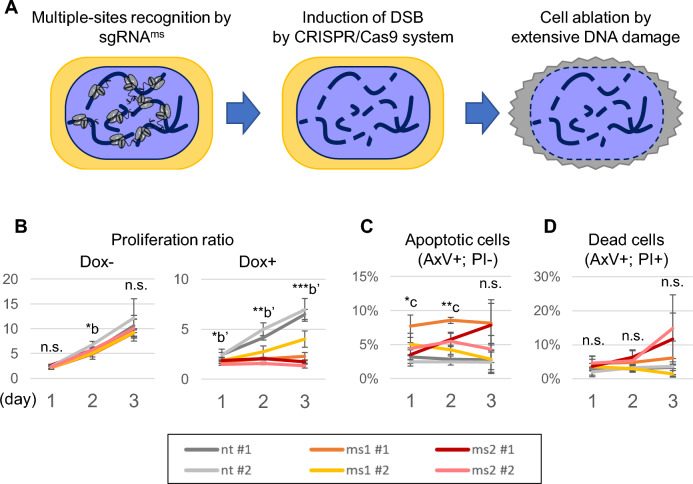
Cell ablation potency of the Cas9-sgRNA^ms^ system in HEK293T. (**A**) The strategy of the Cas9-sgRNA^ms^ system is to induce cell ablation through multiple-site DNA double-strand breaks (DSBs). (**B**) Cell proliferation after Dox-inducible Cas9-sgRNA^ms^ in HEK293T cells. The value of the Y-axis indicates the relative number against day 0 HEK293T cells. (**C**) Early apoptotic cells after Dox-inducible Cas9-sgRNA^ms^ in HEK293T cells. (**D**) Dead cells after Dox-inducible Cas9-sgRNA^ms^ in HEK293T cells. The value of the Y-axis indicates the percentage of AxV+; PI− (**C**) and AxV+; PI+ (**D**). Cas9-sgRNA^nt^ has an empty target recognition sequence in the sgRNA and was used as a control in (**B**–**D**). One-way ANOVA, Tukey honestly significant difference test was used in (**B**–**D**). n.s indicates not significant differences. *b: nt #2 vs ms1 #1 was p < 0.05. *b′: nt #1 or nt #2 vs ms2 #2 were p < 0.05. **b′: nt #1 vs ms1 #2 was p < 0.01, and nt #1 or nt #2 vs ms cell lines except ms1 #2 were p < 0.001. ***b′: nt #1 or nt #2 vs all ms cell lines were p < 0.001. *c: nt #1 or nt #2 vs ms1 #1 were p < 0.01. **c: nt #1 vs ms2 #2 was p < 0.05. nt #1 vs ms2 #1 was p < 0.01. nt #1 vs ms1 #1 was p < 0.001. nt #2 vs ms2 #1 or ms2 #2 were p < 0.01. nt #2 vs ms1 #1 was p < 0.001.

Clustered regularly interspaced palindromic repeats (CRISPR)-Cas9 technology has revolutionized gene editing, whereby DNA double-strand breaks (DSB) can be induced at any desired site in the genome, corresponding to customized single-guide RNA (sgRNAs)^13^. CRISPR-Cas9 is typically used to induce DNA DSBs at single sites for gene editing. Nevertheless, it is hypothesized that programmed cell death can be triggered in cells by inducing multiple DNA DSBs. This technology can kill cancer cells by inducing multiple DNA DSBs in their genome^14,15^ or by targeting cancer-specific fusion oncogenes^16^ without affecting healthy cells. However, this method has not been experimented to induce cell ablation in organ-specific cells to produce organ-deficient animal models. Hence, we aimed to investigate whether organ-deficient animal models could be produced using multiple-target CRISPR-Cas9 (Fig. 1A).

The thymus is the primary organ of the adaptive immune system and is responsible for the education, maturation, and differentiation of immune cells. These immune cells recognize self- and non-self-antigens and are responsible for solid graft rejection in organ transplants^17^. Thymus organogenesis and thymic epithelial cell differentiation are controlled by a single master regulator *Foxn1*^18^ that serves as an ideal candidate for this proof-of-concept study. The athymic, T cell-deficient, viable *Foxn1*^*−/−*^ mouse model^19–21^ can generate a rat PSC-derived thymus via blastocyst complementation^2^. Therefore, we attempted to generate a thymus-deficient mouse model by introducing multiple DSB into Foxn1-expressing cells in this study.

## Results

### Target specificity of the Cas9-sgRNA^ms^ system

Two multiple-site-targeting sgRNAs^ms^ were designed consisting of repeating nucleotide sequences to recognize the Cas9 genome (Table 1). HEK293T cells were co-transfected with the Cas9-sgRNA^ms^-expressing plasmid and the corresponding target DNA sequence of sgRNA^ms^ in the pCAG-EGxxFP plasmid to validate the cleavage activity and target specificity of the Cas9-sgRNAs^ms^ system. EGFP expression indicated the successful cleavage of the target DNA sequence^22^. An EGFP signal was observed in most cells transfected with Cas9-sgRNA^ms1^ or Cas9-sgRNA^ms2^ and pCAG-EGxxFP with the target DNA sequence (Fig. S1). Minimal EGFP signals were detected when sgRNA^ms1^ was used to cleave the ms2 target sequence in pCAG-EGxxFP and vice versa. This further indicated that the targeting of the two sgRNA^ms^ was unique.

**Table 1:** Sequence of sgRNA^ms^ and number of target sites.

sgRNA^ms^	Sequence information (5′–3′)	Number of target site(s)			
		Mouse	Rat	Pig	Human
sgRNA^ms1^	AGGAAGGAAGGAAGGAAGGA	181,903	56,876	21,151	36,251
sgRNA^ms2^	TGGATGGATGGATGGATGGA	22,314	26,853	3,783	12,711

### The Cas9-sgRNA^ms^ system induced multiple DNA double-strand breaks in HEK293T cells

Doxycycline (Dox)-inducible Cas9-T2A-EGFP in HEK293T cell lines that constitutively express sgRNA^ms^ were established to understand the downstream effects of the Cas9-sgRNA^ms^ system and minimize the variation in transfection efficiency between cells (Fig. S2). Two cell lines for each sgRNA^ms^ were used in all subsequent experiments.

Proliferation ratios in the HEK 293T with sgRNA^ms^ were decreased significantly in the presence of Cas9 compared to in the non-targeting (nt) sgRNA control after Dox treatment (Fig. 1B). The cells were stained with annexin V (AxV) and propidium iodide (PI) and analyzed using flow cytometry to further determine whether the cells were undergoing apoptosis and cell death. Early apoptotic cells were defined as AxV^+^ and PI^−^ cells, whereas dead cells were defined as AxV^+^ and PI^+^ cells. We observed a significant increase in the percentage of early apoptotic cells (AxV^+^; PI^−^) at day 2 after Dox treatment, however, day 3 after Dox treatment, dead cells (AxV^+^; PI^+^) did not show significant differences compared to the control (Fig. 1C,D and Fig. S3).

**Figure 2:**
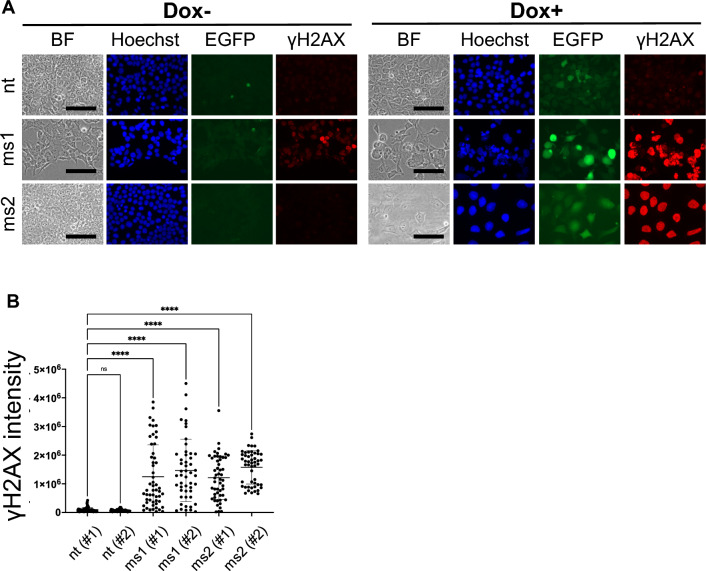
Induction of DNA DSB in HEK293T by the Cas9-sgRNA^ms^ system. (**A**) DNA DSB assays detected γH2AX accumulation. sgRNA^ms^ were driven by the ubiquitously expressing U6-promoter, and Cas9 expression was induced by Doxycycline; the Cas9-sgRNA^ms^ system functioned after adding doxycycline to the culture medium. BF means bright field. Blue: nuclei were stained by Hoechst33342, green: Cas9 expression was monitored by EGFP signal, and red: DNA DSBs were indicated by γH2AX accumulation. Images show data from the nt (#2), ms1 (#2), and ms2 (#2) cell lines. The other cell lines showed the same results. The data were quantitatively analyzed as shown in (**B**). (**B**) Quantitative analysis of γH2AX intensity. One-way ANOVA, Tukey honestly significant difference test was used in (**B**). *p < 0.05; **p < 0.01; ***p < 0.001; ****p < 0.0001. “ns” in (**B**) means not significant differences.

We investigated the DNA DSB after 72 h of doxycycline-induced Cas9 expression. Histone H2AX phosphorylated at serine 139 (γH2AX) accumulates at DNA damage sites and serves as a molecular marker of DNA DSBs^23^. We stained doxycycline-induced cells for γH2AX to detect DNA DSBs as our Cas9-sgRNA^ms^ system was designed to cleave DNA at multiple sites. Cells that expressed sgRNA^ms^ showed a significant increase in γH2AX signal intensity compared to cells expressing non-targeting (nt) sgRNA as a control (Fig. 2A,B). This suggests that the Cas9-sgRNA^ms^ system altered cell growth through DNA DSB but did not induce apoptosis within this time frame.

**Figure 3:**
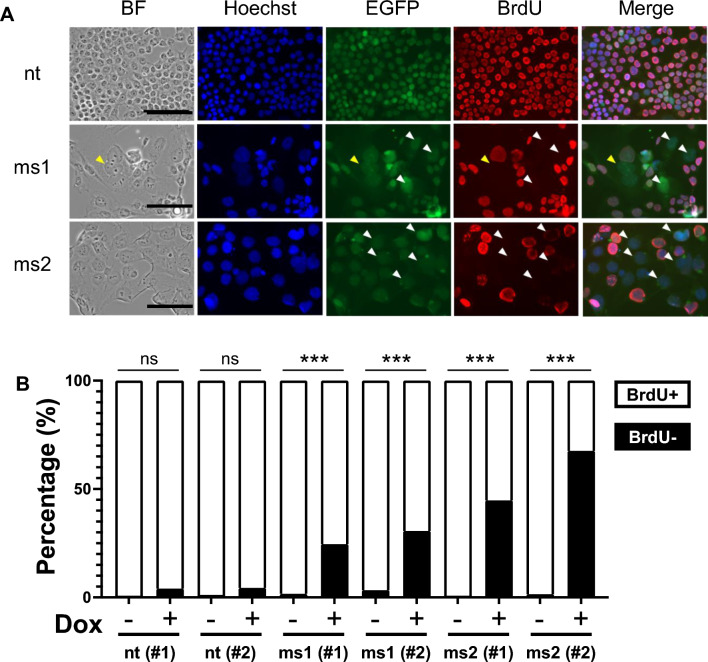
Obstruction of HEK293T cell proliferation by the Cas9-sgRNA^ms^ system. (**A**) Cell proliferation assay with BrdU staining. BF indicates bright field. Blue: nuclei were stained by Hoechst33342, green: Cas9 expression was monitored by EGFP signal, and red: DNA synthesis was indicated by BrdU incorporation. Pictures show data from the nt (#2), ms1 (#2), and ms2 (#2) cell lines treated with Dox. The other cell lines showed the same results. Yellow arrows indicate multiple nuclei and white arrowheads indicate Cas9-expressed BrdU negative cells. The data were quantitatively analyzed as shown in (**B**). (**B**) Quantitative analysis of DNA synthesis via BrdU incorporation. Fisher’s exact probability test was used in (**B**). ***p < 0.001. “ns” in (**B**) means no significant differences.

The cell proliferation rate of cells significantly decreased with the expression of sgRNA^ms1^ and sgRNA^ms2^ compared to the controls using an MTT [3-(4,5-dimethylthiazol-2-yl)-2,5-diphenyltetrazolium bromide] assay to compare cell proliferation in the presence and absence of doxycycline (Fig. S4). The decrease in cell proliferation was negatively affected. Therefore, we hypothesized that DNA synthesis might also be affected by the Cas9-sgRNA^ms^ system. BrdU is incorporated into DNA during DNA synthesis in cells, we performed BrdU staining and showed that there was a significant increase in the proportion of BrdU-negative cells among cells expressing sgRNA from a minimum of 100 cells counted for each cell line (Fig. 3). The nuclei in Cas9-sgRNA^ms^ modified cells were larger than those in the control. Taken together, these results suggest that the designed Cas9-sgRNA^ms^ system induced multiple DNA DSBs throughout the genome and negatively affected HEK293T cell proliferation.

### The Cas9-sgRNA^ms^ system induced cell ablation in mESCs

The aim of this study was to ablate organ-specific cells in mouse models. Therefore, it was necessary to further test whether the Cas9-sgRNA^ms^ system was feasible in mouse cells. The function of mouse ESC lines with sgRNA^ms^ knocked-in the *Rosa26* locus (*Rosa26_ms*) was tested by transfecting Cas9 and a puromycin-resistant gene-expressing plasmid that has no target sequence of sgRNA in PX459. Fewer ALP^+^ cells were detected puromycin selection compared with wild-type mESCs (Fig. S5). This indicated that the simultaneous expression of Cas9 and sgRNA^ms^ induced cell death in most mESCs.

The *p53* tumor suppressor gene is known to play a pivotal role in controlling cell cycle arrest and cell death. Therefore, we further investigated whether cell ablation triggered by Cas9-sgRNA^ms^ was related to the *p53* pathway. There was a significant decrease in the relative number of early apoptotic cells (AxV^+^; PI^−^) and dead cells (AxV^+^; PI^+^) when we transfected Cas9-sgRNA^ms^ into two individual *p53*-KO mESC lines (Fig. S6). These data suggest that Cas9-sgRNA^ms^-induced cell ablation was related to the *p53* pathway.

### *Foxn1*^*Cas9*^; *Rosa26_ms* mice were athymic and lack peripheral T cells

The thymus was selected as the target organ to generate an organ-deficient mouse model using the Cas9-sgRNA^ms^ system. Thymus-deficient mice were reported to be viable until the adult stage, have immunodeficiency owing to a lack of thymus and peripheral mature T cells, and have disrupted hair growth caused by the *Foxn1* gene^19,21^. Furthermore, the thymus consists of PSCs formed by blastocyst complementation using^2^ *Foxn1*^*nu/nu*^.

**Figure 4:**
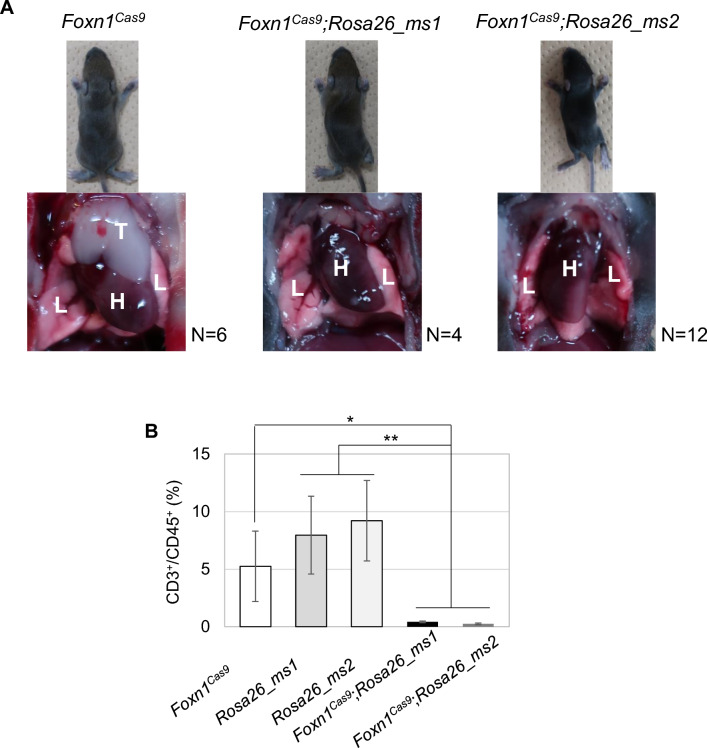
Phenotypes of *Foxn1*^*Cas9*^; *Rosa26_ms* mice. (**A**) Pictures of nine-day old mice. *Foxn1*^*Cas9*^; *Rosa26_ms* mice had an athymic phenotype but were unhealthy. *Foxn1*^*Cas9*^ mice were used as normal phenotype controls. T indicates thymus, L indicates lung, and H indicates heart. (**B**) T cell population in splenocytes determined using flow cytometry. One-way ANOVA, Tukey honestly significant difference test was used in (**B**). *p < 0.05; **p < 0.01.

We established founder mouse lines of *Foxn1*^*Cas9*^ and *Rosa26_ms* from mESCs and crossed with wild-type females to obtain F1 progeny respectively. Each F1 mouse was crossed to obtain the next generation. Next generation pups of *Foxn1*^*Cas9*^; *Rosa26_ms* pups were dissected at 9-day postpartum (P9) to examine the thymus phenotype. *Foxn1*^*Cas9*^; *Rosa26_ms* mice were athymic; however, their hair phenotypes were unaffected (Fig. 4A). Therefore, Epcam-positive cells were collected from the thymus and skin using an Fluorescence Activated Cell Sorter (FACS) and differences in *Foxn1* mRNA expression levels between thymic epithelium and keratinocyte were examine. The results revealed significantly lower *Foxn1* expression in the skin keratinocytes compared to in the thymic epithelium. Furthermore, *Cas9* mRNA expression level in *Foxn1*^*Cas9*^ mice was high in thymic epithelium, similar to Foxn1 expression, while it was low in skin keratinocytes (Fig. S7). The results suggest that the Cas9-sgRNA^ms^ system induced cell ablation depending on Cas9 expression level.

**Figure 5:**
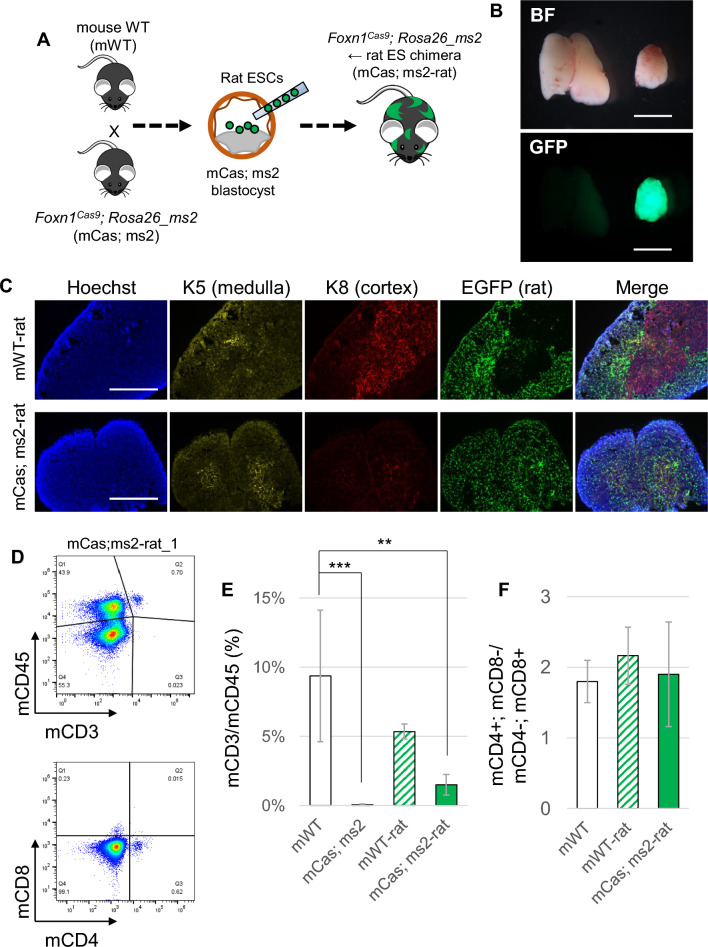
Rat thymus formation using rat ESCs and *Foxn1*^*Cas9*^; *Rosa26_ms* embryos by blastocyst complementation. (**A**) Scheme of generation of rat thymus using the Cas9-sgRNA^ms^ system. (**B**) Thymus in *Foxn1*^*Cas9*^; *Rosa26_ms2* mouse host with rat ESC chimera at day 5 post birth (right). EGFP signals indicate the rat-derived cells. *Rosa26_ms2* mouse littermate was used as controls (left). BF means bright field. (**C**) Thymus sections in interspecies chimeras. Anti-K5 antibody was used to detected mouse and rat medullary thymic epithelial cells and anti-K8 antibody for mouse cortical thymic epithelial cells but not rat cortical thymic epithelial cells. The EGFP signal indicates rat thymic epithelial cells in the interspecies chimera. Hoechst indicates the nucleus. The bars show 2.5 mm in (**B**) and 400 µm in (**C**). (**D**) FCM analysis of splenocytes in *Foxn1*^*Cas9*^; *Rosa26_ms2* mouse host with rESC chimera at day 5 post birth. (**E**) Percentages of the mouse CD3-positive cells in the mouse CD45-positive splenocytes. (**F**) The ratio of mouse CD4 and mouse CD8 in the splenocyte. Wild type mouse (mWT, n = 5), *Foxn1*^*Cas9*^; *Rosa26_ms2* mouse (mCas; ms2, n = 5), wild type mouse host with rat ES chimera (mWT-rat, n = 3), *Foxn1*^*Cas9*^; *Rosa26_ms2* mouse host with rat ESC chimera (mCas; ms2-rat, n = 4) were analyzed in E and F. The other chimeras’ FCM data are shown in Figs. S10 and S11. One-way ANOVA, Tukey honestly significant difference test was used in (**E**) and (**F**). **p < 0.01; ***p < 0.001.

To further analyze the phenotype of thymic deficiency, the splenocytes were analyzed to confirm the presence of T-cell populations in peripheral blood. *Foxn1*^*Cas9*^; *Rosa26_ms* mice had significantly fewer peripheral CD3^+^ and CD45^+^ cells in the spleen compared to control mice lacking the Cas9-sgRNA^ms^ system according to immunostaining and flow cytometry (FCM) (Fig. 4B). Additionally, the CD4/CD8 ratio is one of biomarkers for immunological dysfunctions such as immunodeficiency and autoimmunity; among the in wild type, *Foxn1*^*Cas9*^, *Rosa26_ms1*, and *Rosa26_ms2* mice splenocytes there were no significant differences the CD4/CD8 ratio (Fig. S8 and Fig. 5F). The Cas9-sgRNA^ms^ system thus successfully generated a thymus-deficient mouse model.

### Generation of rat thymus in the interspecies chimera using the Cas9-sgRNA^ms^ system

Rat ESCs (rESCs) tagged with EGFP were injected into mouse blastocysts that were obtained from wild type mice crossed with *Foxn1*^*Cas9*^; *Rosa26_ms* mice, followed by transfer to pseudo pregnant female mice to continue embryo development (Fig. 5A). 137 chimeric embryos were transferred into seven pseudopregnant mice, resulting in 44 offspring. Among them, 24 were interspecies chimeras carrying rat cells labeled with EGFP. Five interspecies chimeras carrying *Foxn1*^*Cas9*^; *Rosa26_ms2* genotype in host mouse cells survived until day five post-birth (P5), and four of them had formed thymus. The thymus had high EGFP expression (Fig. 5B). However, the morphology of the complemented thymus was slightly different from that of the control thymus, which was used as a host for the non-working Cas9-sgRNA^ms^ system for genotyping. Therefore, we analyzed the thymus sections to understand the contribution of rESC-derived cells. The thymus of the control host contained partially rat-derived thymic epithelial cells. In contrast, the thymic epithelial cells from the *Foxn1*^*Cas9*^; *Rosa26_ms* host were mostly derived from rESCs (Fig. 5C). Immunostaining of these thymi with cortex (K8)-and medullary (K5)-specific markers showed that rESCs contributed to the cortical and medullary regions in the thymus (Fig. 5C). The cortex marker (K8) used in this study could only detect mouse tissue, not rat tissue (Fig. 5C and Fig. S9). Peripheral blood T-cell populations were analyzed to assess the functionality of the generated rat thymus. The presence of CD3-positive cells as well as CD4^+^; CD8^−^ and CD4^−^; CD8^+^ cells was observed. The CD3-positive population in the Foxn1Cas9; Rosa26_ms mouse host with rES chimera, which had rat thymus was significantly lower than that in the wild type at P5; however, CD4/CD8 ratio showed no significant difference (Fig. 5D–F, Figs. S10, and S11). Overall, the data suggest that *Foxn1*^*Cas9*^; *Rosa26_ms* mice create an empty thymus niche that can be complemented with injected rESCs.

## Discussion

The in vitro and in vivo data showed that the designed Cas9-sgRNA^ms^ system induced cell ablation through DNA DSB induction, cell apoptosis, and cell proliferation defects, and eventually led to complete ablation of the thymus. An inducible Cas9-EGFP HEK293T cell line experiment showed that Cas9-sgRNA^ms^ expression generated high levels of γH2AX and significantly increased the percentage of BrdU-negative cells. This indicated that the Cas9-sgRNA^ms^ system induced the accumulation of DNA DSB and prevented cell proliferation. Furthermore, almost all ms1 and ms2 cells showed changes in nuclear morphology following Cas9 expression (Figs. 2A and 3A). In vitro data showed only a low percentage of apoptotic cells after Cas9 expression; therefore, it is possible that more cell death occurred at a later stage in the Cas9-sgRNA^ms^ system. Alternatively, they may induce cellular senescence because there are some reports that nuclear morphology (such as giant nuclei and multinuclei) and cell cycle arrest can be a biomarker of senescent cells^24–26^. Some reports show that senescent^27,28^ and apoptotic cells^29,30^ can be phagocytosed in vivo. Foxn1-expressed thymic epithelium may have been removed by phagocytosis after working with the Cas9-sgRNA^ms^ system.

*Foxn1*^*nu/nu*^ mice exhibit a hairless and athymic phenotype^19,20^. However, only the thymus was affected in the *Foxn1*^*Cas9*^; *Rosa26_ms* mouse model, and not the hair phenotype. Foxn1 is also expressed in developing keratinocytes in the skin, and plays a role in hair follicle and epidermal development^31,32^. We then compared *Foxn1* expression levels in thymic epithelium and keratinocytes of the skin. The expression level in keratinocytes of the skin was significantly lower than that in thymic epithelium, and was correlated with *Cas9* expression level (Fig. S7). The result suggested that cell ablation by the Cas9-sgRNA^ms^ system depended on promoter activity, namely, Cas9 expression level.

Particularly, since Foxn1 expression stimulates keratinocyte growth in neighboring cells via paracrine mechanisms^32^, it is possible that even a small number of Foxn1-expressing cells remaining in the skin can maintain normal growth and differentiation of hair follicles and keratinocytes. Therefore, there may be incomplete or partial ablation of Foxn1-expressing cells in *Foxn1*^*Cas9*^; *Rosa26_ms* mouse skin.

The thymus derived from rESC showed a high contribution (strong EGFP signal) to *Foxn1*^*Cas9*^; *Rosa26_ms* mouse hosts (Fig. 5). The same observation was consistent with previous reports of the generation of a rat thymus in a nude mouse model^2^.

The entire thymus section lacked mouse cTECs in the chimera at E18.5. However, the absence of mouse mTECs could not be proved since the marker of mTEC (anti-Keratin 5 antibody) recognized both mouse and rat Keratin 5 (Fig. 5C). A study on embryonic thymus development tracking the expression of β5t (a proteasome subunit specifically expressed in cTEC but not in mTEC) showed that almost all Aire+ mTECs had a history of β5t expression^33^. Other studies show that mTECs differentiate from cells that previously expressed cTEC markers such as CD205^34^ and IL7^35^. These reports suggested that thymic progenitor cells first differentiate into cTECs and then into mTEC to form a mature thymus. Furthermore, it is unlikely that mouse cTECs preferentially differentiated into mTECs since mouse cTECs were extensively detected in the interspecies chimeric thymus (Fig. 5C). Subsequently, mouse cTEC were not detected in the thymus produced with *Foxn1*^*Cas9*^; *Rosa26_ms* mouse and rat ESCs. Taken together, these results suggest that most of the thymic epithelium in the thymus produced from *Foxn1*^*Cas9*^; *Rosa26_ms* mouse and rat ESCs was derived from rESCs.

The functionality of the generated rat thymus at P5 revealed a lower proportion of T cells in the peripheral blood that in the wild-type mice. T cells undergo education in the thymus after birth and circulate into the peripheral blood. The size of the rat thymus in the chimera was smaller than that of littermate mice (Fig. 5B), as reported previously^2^, suggesting that the number and surface area of thymic epithelial cells may influence the number of T cells in the peripheral blood shortly after birth. Additionally, the thymus serves as a site for educating self/non-self discrimination, where immature T cells that are mismatched undergo apoptosis. It is possible that rat thymic epithelium imposes stricter selection on immature T cells from mice than between mice themselves. Further detailed investigations would reveal more insights in future.

Cas9 was expressed under organ-specific conditions to obtain an organ-deficient animal model with constitutive expression of sgRNA^ms^. This could be potentially applied to generate animals with deficiencies in different organs and is hypothesized to ablate actively proliferating progenitor cells. The sequence of sgRNA^ms^ designed in this study has many targeting sites in the genome of several species; thus, it can be applied to generate organ-deficient mouse models and larger animal models (such as pigs) to procure human organs. The efficiency of the Cas9-sgRNA^ms^ system with the gene KO method to generate organ-deficient animal models increased from one-quarter to one-half when the Cas9-sgRNA^ms^ system was used. This can be achieved by crossing animals with a heterozygous Cas9 knock-in with another animal with a homozygous *Rosa26_ms*. This system remains an issue since the induction of the organ-deficient phenotype depends on the developmental mechanisms of the target organ. However, this system has a different mechanism of induction of cell ablation compared to the KO animal model. In particular, the Cas9-ms system is potentially influenced by promoter activity. It may be possible to avoid abnormalities in organs other than the intended target, which has been a challenge with previous KO or DTA systems, by using the Cas9-ms system. In future, monitoring promoter activity with knock-in models using marker proteins such as GFP may lead to higher specificity for the organs intended for disruption.

## Method

### Animals

Animal handling, breeding, and all experimental procedures were conducted under specific-pathogen-free (SPF) conditions at the Nara Institute of Science and Technology (NAIST), according to the guidelines of “Regulations and By-Laws of Animal Experimentation at the Nara Institute for Science and Technology”, and were approved by the Animal experimental Committee at the Nara Institute of Science and Technology (the approval no. 1639 and no. 2103). Study of the animal experiments were carried out in compliance with the ARRIVE guidelines^36^. ICR, C57BL6/J, and B6D2F1 mice used in this study were purchased from SLC, Japan, and maintained in the NAIST Animal Facility. The mouse is maintained under 12 h light/12 h dark cycle (light on 08:00 AM and light off 08:00 PM). Female ages are 8–12-week-old for generating chimeras and over the 8-week-old for breeding. Male vasectomized mice are prepared by cutting their vas deferens. Food and water are available ad libitum. For the collection of eggs and organs, mice and interspecies chimeric animals euthanized by cervical dislocation. For the embryo transfer to uterus, pseudopregnant female mice were operated under the three drug-mixed anesthesia, 0.5 mg/kg of dexmedetomidine hydrochloride (DOMITOR: ZENOAQ), 4.0 mg/kg of midazolam (Midazolam Injection: SANDOZ), and 5.0 mg/kg of butorphanol tartrate (vetlf5: Meiji Animal Health Co., Ltd.). After the embryo transfer, female mice were treated atipamezole hydrochloride (ANTISEDAN: ZENOAQ) via 0.75 mg/kg of subcutaneous injection to wake up from the anesthesia.

### Cell lines

HEK293T, derived from human embryonic kidney cells with SV40 Large T antigen, was cultivated in Feeder Medium, which contained 10% FBS (biosera, S00HA 10004), DMEM (Nacalai Tesque, 08459-64), L-glutamine (Thermo Fihser, 25030081), sodium private (Thermo Fihser, 11360070), Non-Essential Amino Acids (Thermo Fihser, 11140050), and penicillin-streptomycin (Thermo Fihser, 15140122). It is known that HEK293T has high transfection efficiency. Moreover, the target sites of sgRNA^ms^ in the human genome are fewer than those in the mouse genome. Therefore, we considered HEK293T suited for in vitro experiments to assess the effect of DSB by the Cas9-sgRNA^ms^ system. Mouse ESC line was cultivated in N2B27-a2i medium^37,38^. Rat ESC line was cultivated in N2B27-2i medium^39,40^.

### Plasmid constructions

pSpCas9(BB)-2A-Puro (PX459) V2.0 was purchased from Addgene (plasmid #62988)^41^. sgRNAs^ms^ and *p53* targeting sequences were ligated at the BbsI site of PX459 to produce PX459-sgRNA^ms1^ and PX459-sgRNA^ms2^. The EGFP cassette was replaced the puromycin resistance gene (Puro^r^) on the C-terminus of Cas9 in PX459 to produce pCas9-T2A-EGFP-sgRNA^ms1^ and pCas9-T2A-EGFP-sgRNA^ms2^.

sgRNAs^ms^ targeting sequences with PAM were inserted in multi-cloning-site of pCAG-EGxxFP (kindly provided by Dr. M Ikawa at Osaka University (Addgene plasmid #50716)).

pPB-TetOPuro-Cas9-EGFP plasmid for in vitro assay was produced from a Tet-One^TM^ doxycycline (Dox)-inducible system (Cat no. 634303, Takara Bio Japan) with some modifications. hPGK promoter was replaced to CAG promoter, *T2A-Puro*^*r*^ cassette was inserted into the C-terminus of Tet-On 3G (*TetOn3G- T2A-Puro*^*r*^), and *Cas9-T2A-EGFP* cassette was inserted under TRE3Gs promoter. This construction was inserted between two inverted terminal repeats (ITRs) of PB5 and PB3 by replacing the CAG.OKSML-puDtk of pPB- CAG.OKSML-puDtk (kindly provided by Dr. K Yusa at Sanger Institute)^42^ to produce pPB-TetOPuro-Cas9-EGFP (Fig. S2A).

pHyg-sgRNA^ms^ plasmids were produced using PX459 and pcDNA4-TO-Hygromycin-mVenus (Addgene plasmid #44099). *SV40 promoter-Hyg*^*r*^*-SV40 poly (A) signal* cassette in pcDNA4-TO-Hygromycin-mVenus was inserted and replaced to *Cbh promoter-Cas9-T2A-Puro*^*r*^*-bGH poly (A) signal* in PX459.

Targeting vectors for knock-in at *Foxn1* and *Rosa26* were constructed on the pLSODN-4D (Biodynamics Laboratory Inc.) backbone, consisting of a gene of interest flanked by left and right homology arms. The primers used for homologous arm construction are listed in Supplementary Table S1. Knock-in strategies were shown in Fig. S7.

CRISPR/Cpf1 system was used for knock-in of hU6-sgRNA^ms^ cassette and Cas9 cassette at *Rosa26* and *Foxn1*. The crRNA used for Cpf1 recognition sites were ligated to BsmBI site of Cpf1-expressing vector, pTE4398 (Addgene plasmid #74042)^43^. All Cpf1-crRNA target sequences were listed in Supplementary Table S2.

The number of sgRNAs^ms^ targeting sites, and targeting sites for *p53*, *Foxn1* and *Rosa26* were defined using CRISPRdirect software^44^.

### Generation of the Dox-inducible *Cas9-sgRNA*^*ms*^ HEK293T cell lines

To generate the Dox-inducible *Cas9-sgRNA*^*ms*^ HEK293T cell lines, pPB-TetOPuro-Cas9-EGFP, and pCMV-hyPBase plasmid (kindly provided by Dr. K Yusa at Sanger Institute) were co-transfected using PEI-MAX (Polysciences) into HEK293T cells and selected with puromycin (0.5 µg/mL) for 7 days to establish cell lines (Fig. S2B,C). Subsequently, pHyg-sgRNA^ms^ were transfected into the parental Dox-inducible Cas9-EGFP HEK293T cell line, then the Dox-inducible *Cas9-sgRNA*^*ms*^ HEK293T cell lines were established through hygromycin B (300 µg/mL) selection for 7 days.

### Generation of *p53-KO* mouse ESC lines

Mouse ESCs, mF1-05 from 129X1/B6J F1^38^, were transfected with the sgRNA set for mouse *p53* knockout and selected using 1 µg/mL puromycin for 3 days. PCR-based genotyping and karyotyping were conducted to establish *p53*^*−/−*^ cell lines. sgRNAs target sites and PCR primers were listed in Supplementary Tables S2 and S3, respectively.

### Establishment of *Foxn1*^*Cas9*^ and *Rosa26_ms* mouse lines

Both the Cpf1-expressing plasmid and targeting vector for knock-in were transfected into mouse ESCs (mF1-05) using Lipofectamine 3000 (Thermo Fisher). G418 (150 µg/mL) selection was conducted for 3 days in a2i/L media^37^, colonies were picked at ten days after transfection, and screened for knock-in through genotyping and sequencing. All primers used for genotyping are listed in Supplementary Table S3.

Established mESC lines were used to generate chimeric animals. Female ICR mice aged 8–10 weeks were administered with 0.1 mL of CARD HyperOva^®^ (KYUDO COMPANY) through intraperitoneal injection. After 48 h, hCG (7.5 IU) was administered through intraperitoneal injection. The superovulated female ICR mice were mated with male ICR mice. The presence of a vaginal plug the following morning indicated the occurrence of coitus and was defined as E0.5. E1.5 embryos were collected by flushing M2 media (Sigma) through the oviducts. Embryos were cultured at 37 °C, 5% CO_2_ condition in KSOM media^45^ until use.

Cultured embryos at the 8-cell stage were injected with mESCs using a piezo-micromanipulator (PMAS-CT150, Prime Tech LTD) under a light microscope (DM-IRC-Leica). Each embryo was injected with six to eight mESCs in M2 medium. The injected embryos were cultured until the blastocyst stage (E3.5) in KSOM medium and transferred to the uterus of E2.5 pseudopregnant mice under anesthesia. Chimeras were recovered by natural delivery or Caesarean section on E19.5. To generate F1 mouse lines, male chimeras were mated with C57BL6/J females or B6D2F1 females. The genotype of F1 mice was identified by PCR and sequencing.

### Generation of the interspecies chimera for blastocyst complementation

Rat ESCs tagged with EGFP, rG104 from Wistar/F344 F1 (kindly provided by Dr. Ikawa at Osaka University)^40^, were injected into mouse blastocysts that were obtained from super ovulated *Rosa26_ms* females crossed with *Foxn1*^*Cas9*^; *Rosa26_ms* males. Embryo collection and transferring to pseudo pregnant female mice were performed the same method as generating chimera. Generated interspecies chimeras were identified by EGFP signal derived from rat cells under a fluorescent microscope (MZFL III, Leica). The mouse host genotype of chimera was identified by PCR.

### Flow cytometry analysis

HEK293T cells stained Alexa Fluor 647-conjugated annexin V, 1:50 (BioLegend) and propidium iodide (1 µg/mL) in DMEM (Nacalai Tesque, 08459-64) with 10 mM HEPES on room temperature for 15 min. Samples were filtered through a 37 µm mesh prior to cytometry analysis.

Splenocytes were collected from adult and P9, P5 mice, and P5 interspecies chimeras by mashing the spleen between glass slides and chilling it in PBS(–). Splenocytes were stained with APC anti-mouse CD45 (1:50, Biolegend 100516), PE anti-mouse CD3ε (1:50, Biolegend 100308), Alexa Fluor 488 anti-mouse CD3ε (1:50, Biolegend 100321), APC anti-mouse CD4 (1:50, Biolegend 100516), PerCP anti-mouse CD8 (1:50, Biolegend 100732), APC anti-rat CD45 (1:50, Biolegend 202212), PE anti-rat CD3ε (1:50, Biolegend 201412), APC anti-rat CD4 (1:50, Invitrogen 17-0040-82), and PerCP anti-rat CD8 (1:50, Biolegend 201712) in 50 µL of 0.1% BSA/PBS at 4 °C for 30 min. The samples were resuspended in 500 µL of 0.1% BSA/PBS and filtered through a 37 µm mesh prior to flow cytometry analysis.

Flow cytometry was conducted using a BD Accuri C6 flow cytometer (BD Biosciences), and data were analyzed using FlowJo software (Becton Dickinson).

### MTT assay

Inducible Cas9-sgRNA^ms^ HEK293T cells were seeded at a density of 5 × 10^3^ cells/well in 96 well plates coated with 0.2% Matrigel in the basal media. The cells were induced with doxycycline (1.5 µg/mL) for 72 h, and an MTT cell count kit (Nacalai Tesque, 23506-80) was used to perform the MTT assay. Cells were treated with 10 µL of MTT (3-(4,5-dimethylthiazol-2-yl)-2,5-diphenyltetrazolium bromide) tetrazolium solution and incubated for 3 h at 37 °C, followed by adding 100 µL of solubilization solution and overnight incubation at 37 °C. The absorbance at 595 nm was measured with a reference reading at 620 nm using a multi-mode plate reader (Berthold, TriStar LB942). The cell proliferation rate was calculated using the following formula:

$$Cell\,\, proliferation\,\, rate \left(\%\right)= \frac{{A}_{595} {Dox}^{+}}{{A}_{595} {Dox}^{-}} \times 100\%$$

### DNA synthesis assay

Doxycycline-inducible Cas9-sgRNA^ms^ HEK293T cells were cultured in 6-well plates and attached to glass coverslips. After 48 h of doxycycline induction, BrdU was added to the culture at a final concentration of 10 µM and further incubated for another 24 h. The cells were briefly washed in cold phosphate-buffered saline (PBS (–)) (Nacalai). 1 mL 1× cytofix/cytoperm buffer (3.6% PFA and 0.2% saponin) in PBS was added to the cells and incubated at room temperature for 30 min. Buffer was removed and cells were washed once with 1 mL of wash buffer/PBS (5% FBS and 0.2% saponin). 1 mL of cytoperm plus/PBS buffer (5% BlockAce (KAC Co., Ltd.) solution, and 0.5% Triton-X 100) was then added and incubated on ice for 10 min. The buffer was then removed, and added with 1 mL of 1× cytoperm/cytofix buffer was added and incubated at room temperature for 5 min. The buffer was removed and washed with 1 mL of wash buffer. Coverslips were gently removed from the 6-well plates with fine-tipped forceps and cells were treated with 50 µL of 0.3 mg/mL DNaseI in PBS at 37 °C for 1 h. The cells were washed twice with the wash buffer at room temperature. Anti-BrdU antibody (1:200, Oxford Biotechnology Ltd., OB0030) was reacted on ice for 30 min. The cells were washed in wash buffer three times, Alexa Fluor 555 conjugated anti-rat IgG (H+L) antibody (Thermo Fisher, A21434) was reacted on ice for 20 min. After washing in wash buffer three times, the nuclei were stained with Hoechst33342 (1:1000; KV072, Wako) for 3 min before imaging.

### Alkaline phosphatase (ALP) staining

ALP staining was performed using a Histofine alkaline phosphatase staining kit (Nichirei Bioscience, Japan) according to the manufacturer’s protocol. Cultured cells were washed twice in PBS and fixed by 4% paraformaldehyde (PFA) for 5 min. Freshly prepared ALP staining reagent was added and reacted at room temperature for 30 min. The cells were washed with double-distilled water prior to imaging with a digital camera (COOLPIX P7100, Nikon) or observation under a stereomicroscope.

### Immunostaining

Cells attached to a glass coverslip or tissue section in O.C.T. compound (Sakura Finetek) were used for immunostaining. Tissue sections were prepared at 10 µm thickness using a cryostat (NX70, Leica) and dried at 37 °C for 10 min. The samples were washed twice in 2 mL PBS (–) and fixed in 2% paraformaldehyde (Nacalai Tesque) for 5 min. The samples were then dehydrated with acetone (Nacalai Tesque) for 5 min. Permeabilization was performed in 50 µL 0.5% Triton X-100 (Nacalai Tesque) for 5 min. Samples were then blocked with 10% goat serum (143-06561, Wako) in BlockAce solution (KAC Co., Ltd.) at room temperature for 1 h, followed by washing in 0.1% BSA/PBS (Sigma) twice for 5 min each. Primary antibody (for DNA DSBs analysis, anti-γH2AX, 1:50, Biolegend, 613402; for immunohistochemistry of thymus, anti-K8, 1:50, Biolegend, 904804, and anti-K5, 1:50, Biolegend, 905504) was incubated at 4 °C overnight under humid conditions. The secondary antibody (Alexa Fluor 555 conjugated anti-mouse IgG, Thermo Fisher, A21425; Alexa Fluor 647 conjugated anti-rabbit IgG, Thermo Fisher, A21246) was incubated at room temperature for 1 h. Nuclei were stained with Hoechst33342 (1:1000; KV072, Wako) for 3 min before imaging with a fluorescent microscope (EVOS^®^ FL, Invitrogen). Intensity of γH2AX signals were accessed using ImageJ software^46^.

### Collection of Epcam-positive cells in the skin and thymus using FACS sorting

Embryonic day 16.5 thymuses and skins, and day five post birth skins were digested in 1 mg/mL collagenase (Wako, 037-17603) in DMEM (Nacalai Tesque, 08459-64) at 37 °C for 15 min, and then in 0.25% trypsin in 1 mM EDTA-PBS at 37 °C for 5–10 min. After centrifuging, cell pellets were resuspended to Feeder Medium and filtered through a 37-µm mesh. Samples were stained with Alexa Fluor 647 anti-mouse CD326 (Ep-CAM) (1:100, Biolegend 118212) and PerCP anti-mouse CD45 (1:100, Biolegend 103130) at 4 °C for 30 min. An MA900 Multi-Application Cell Sorter (Sony) was used to collect Epcam-positive and CD45-negative cells (10,000 cells/batch).

Since the E16.5 thymic samples were small in size and composed of a low number of thymic epithelium cells, five thymuses were pooled together as one batch.

### Quantitative RT-PCR analysis

Total RNA was purified using Trizol reagent (Thermo Fisher Scientific) and used for reverse transcription (RT). cDNAs were prepared using the SuperScript IV VILO master mix (Thermo Fisher Scientific). Luna Universal qPCR Master Mix (New England Biolabs) was used to amplify the DNA fragment, and amplified DNA was detected on a LightCycler 96 (Roche). Species specificity of all primer sets was assessed by the amplification curve and melting curve from the qPCR result.

The primers used for RT-PCR are described in Supplementary Table S4.

### Statistical analysis

Statistical tests were performed using Prism 9 (GraphPad) or EZR software^47^. The confidence interval was set at 95%, and a *p-*value less than 0.05 indicates a significant difference between the compared datasets.

### Supplementary Information

Supplementary Information.

## Data Availability

The data that support the findings of this study are available from the corresponding author upon reasonable request.
