# Supplementary material for: Generating an organ-deficient animal model using a multi-targeted CRISPR-Cas9 system
Source: Sci Rep. 2024 May 9;14:10636. doi: 10.1038/s41598-024-61167-3 (PMC11082136; doi:10.1038/s41598-024-61167-3)
Supplement: Supplementary file 1 — Supplementary Information. [file 41598_2024_61167_MOESM1_ESM.pdf]

Title: **Generating an organ-deficient animal model using a multi-targeted CRISPR-Cas9 system**

Jonathan Jun-Yong Lim[1, 3, #], Yamato Murata[1, #], Shunsuke Yuri[1], Kohei Kitamuro[1], Taro Kawai[2], and Ayako Isotani[1]*

1. Laboratory of Organ Developmental Engineering, Division of Biological Science, Graduate School of Science and Technology, Nara Institute of Science and Technology, 8916-5 Takayama-cho, Ikoma, Nara, 630-0912 Japan
2. Laboratory of Molecular Immunobiology, Division of Biological Science, Graduate School of Science and Technology, Nara Institute of Science and Technology, 8916-5 Takayama-cho, Ikoma, Nara, 630-0912 Japan
3. Department of Physiology, Yong Loo Lin School of Medicine, National University of Singapore, Singapore.

# Equal contribution of authors

***Correspondence: isotani@bs.naist.jp**

Figure S1

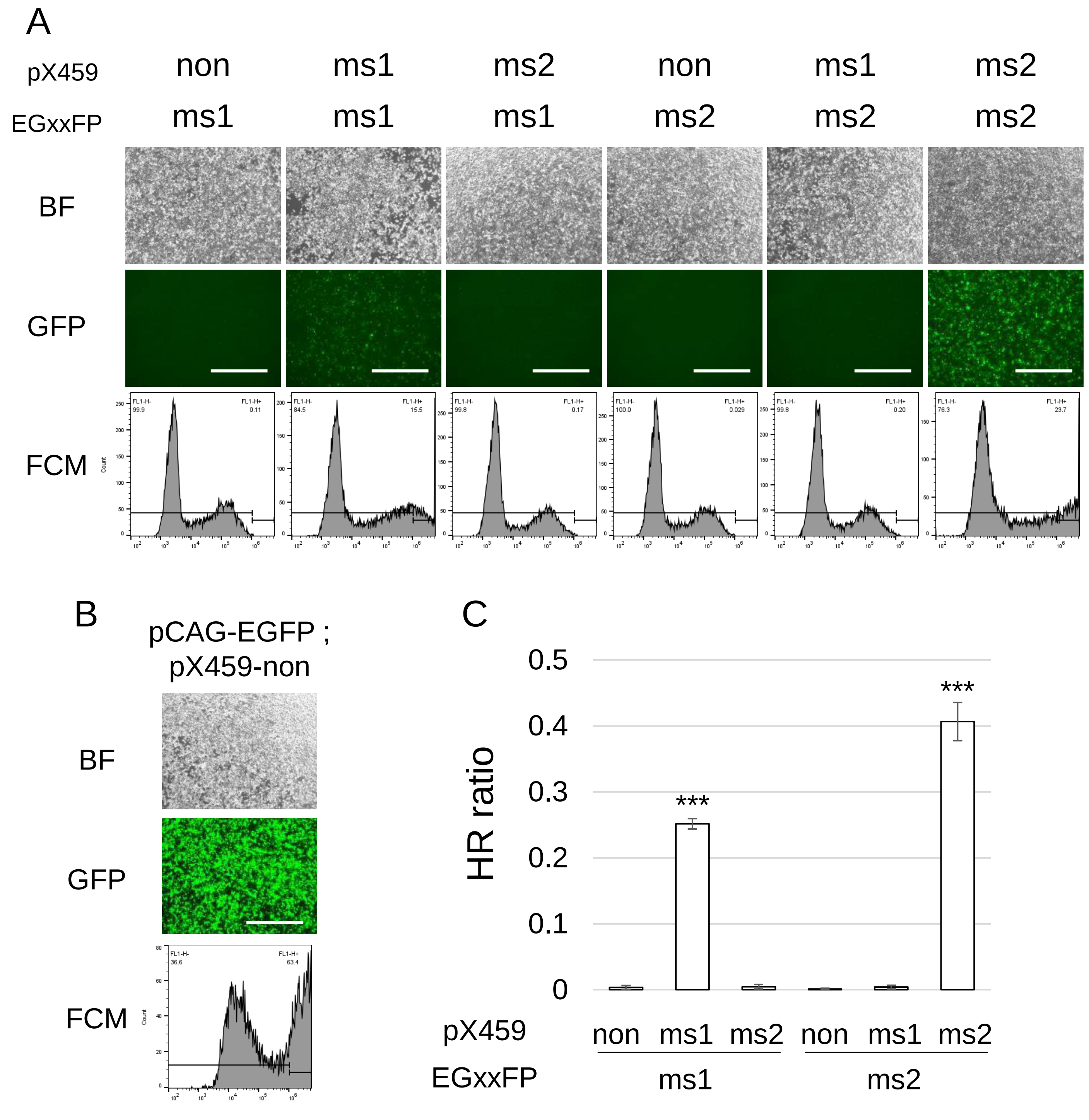

Figure S1. Validation of sgRNAms sequence specificity in HEK293T

(A) HEK293T cells were transfected with Cas9-sgRNA^{ms} and matching sgRNA ms-target or non-matching sgRNA-target vectors. Green signals indicate that the sgRNA target in pCAG-EGxxFP was cleaved by the Cas9-sgRNA^{ms}system; subsequently, it occurred as a homology-dependent repair and then reconstituted the EGFP expression cassette. Scale bars indicate 1mm. The percentage of EGFP-expressed cells was identified by flow cytometry (FCM). (B) pCAG-EGFP and Cas9-sgRNA^{non} vectors were transfected to HEK293T to monitor the transfection efficiency. (C) The homologous recombination (HR) ratios in A were calculated by dividing the GFP-positive percentages for each condition from the GFP-positive percentages for the pCAG-EGFP; pX459-non condition in B. one-way ANOVA, Tukey honestly significant difference test was used in B. *** $p < 0.001$ were showed the other conditions. Experiments were conducted in triplicates.

Figure S2

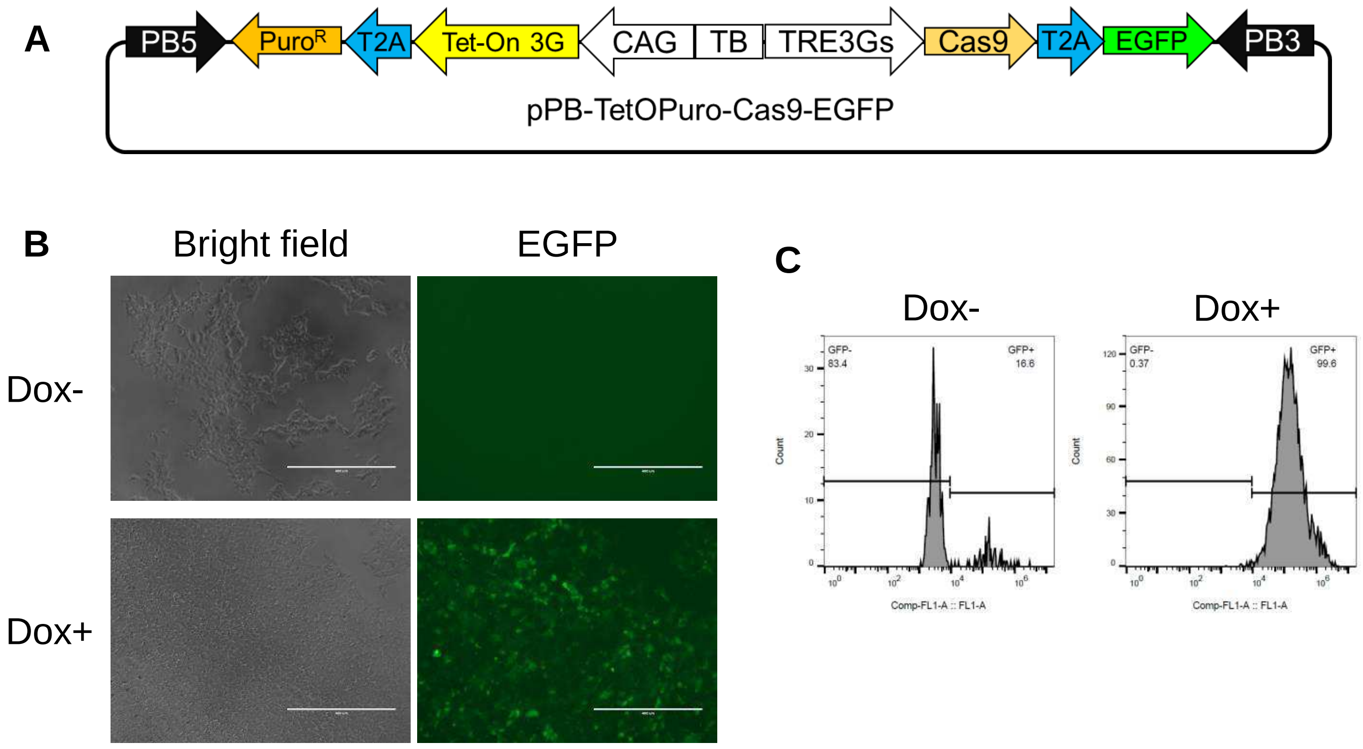

Figure S2. Doxycycline inducible Cas9 in HEK293T

(A) Construction of a doxycycline (Dox)-inducible Cas9-T2A-EGFP vector.

(B) and (C) monitoring Cas9 expression by EGFP signaling after Dox induction.

Dox- indicates no Dox in the culture medium, whereas Dox+ indicates Dox in the culture medium. Bars show 400 μm in B.

Lim JJY et al.,

Figure S3

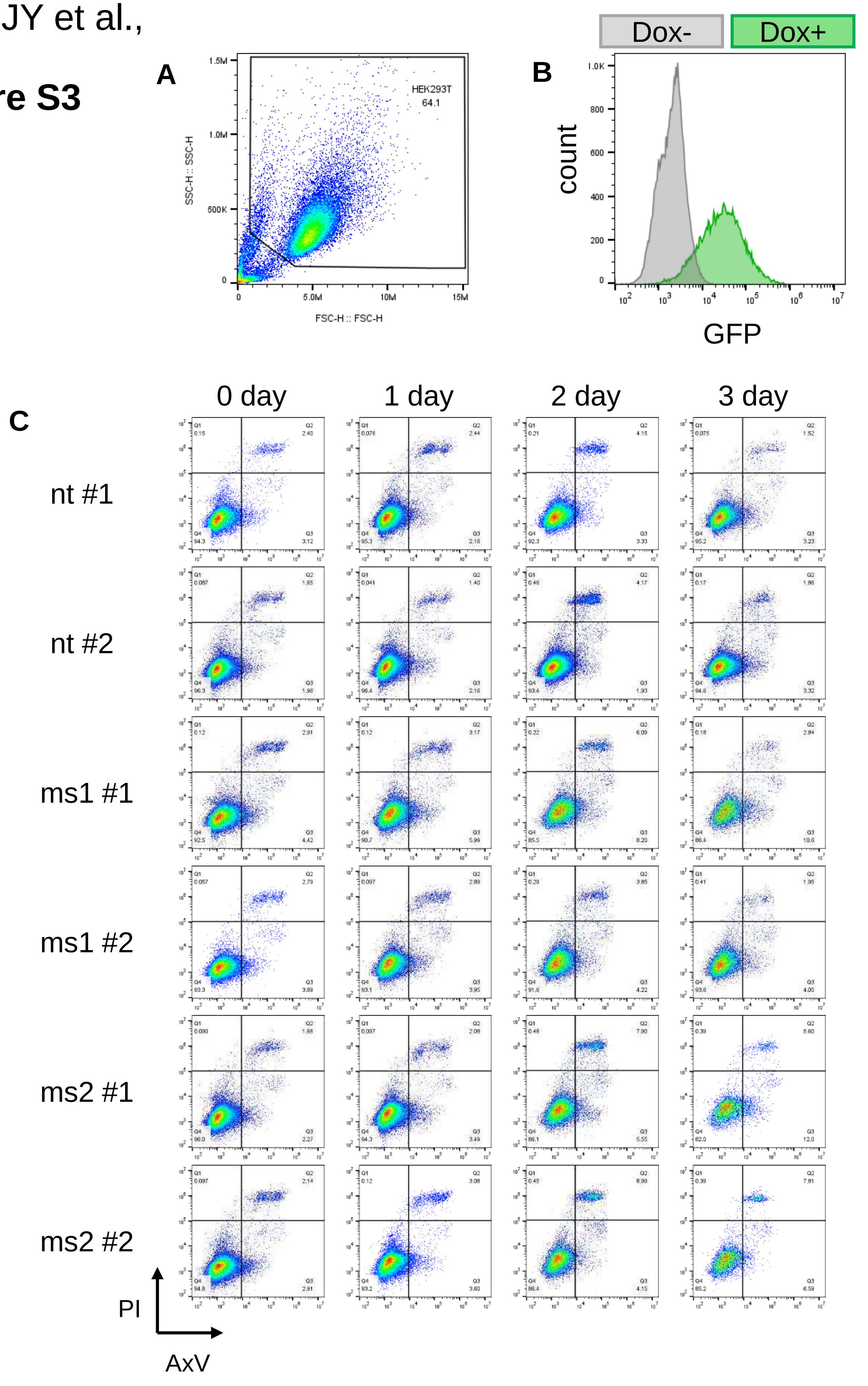

Figure S3. FCM analysis for detecting apoptotic cells and dead cells in HEK293T cells with the Dox inducible Cas9-gRNAms system. (A) Gates of the HEK293T area for this assay. (B) EGFP expressed cells of Dox with or without after 72 hours in the nt #1 cell line. (C) Sequential analysis of apoptotic cells (AxV+; PI-) and dead cells (AxV+; PI+). Experiments were conducted in triplicates.

Lim JJY et al.,

Figure S4

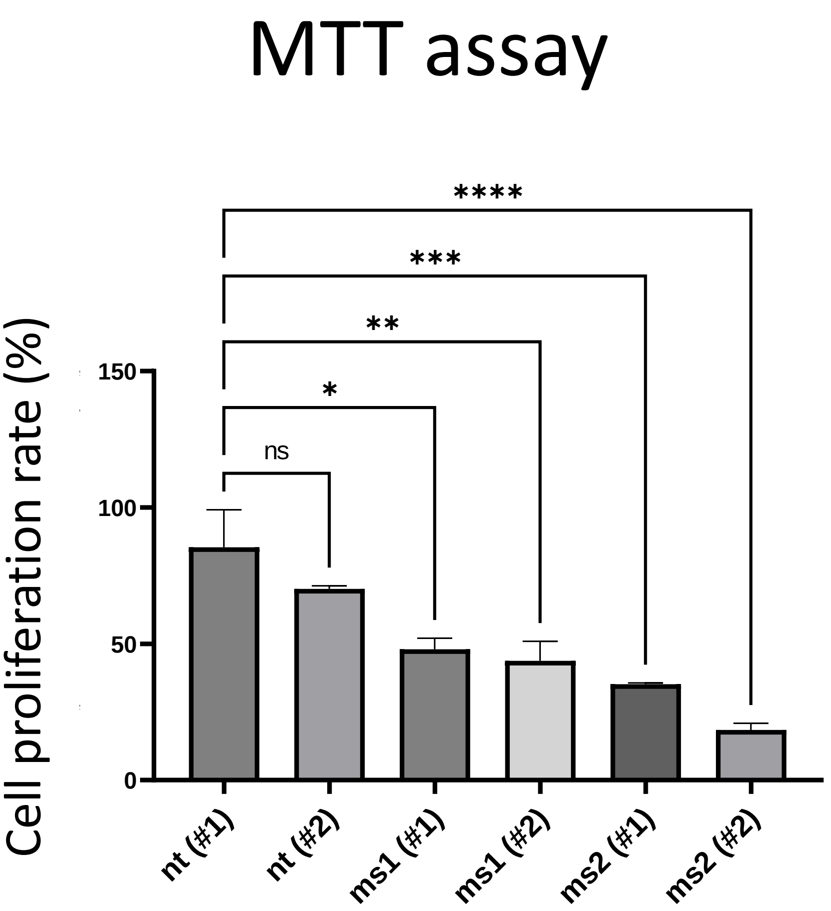

Figure S4. Cell proliferation assay in HEK293T using the MTT assay
The proliferation rate of HEK293T cells expressing sgRNA^{ms1} (ms1), sgRNA^{ms2} (ms2), and non-targeted sgRNA (nt) after 72 h of DOX-induced Cas9-EGFP expression. One-way ANOVA, Tukey honestly significant difference test was used for statistical analysis. *p<0.05; **p<0.01; ***p<0.001; ****p<0.0001. Experiments were conducted in triplicates.

Lim JJY et al.,

Figure S5

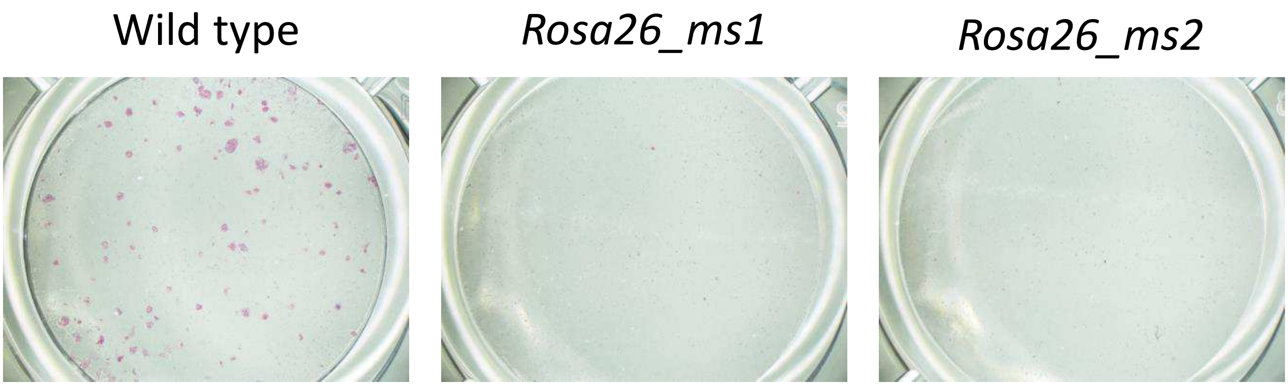

Figure S5. Effects of Cas9-sgRNA^{ms} system in mouse ESCs
Colony formation after transient Cas9 expression in the mouse ESC line, which has a ubiquitously expressed sgRNA^{ms} cassette knocked-in the *ROSA26* locus.
Alkaline phosphatase-positive colonies are shown in light violet and represent surviving mESCs.

Lim JJY et al.,

Figure S6

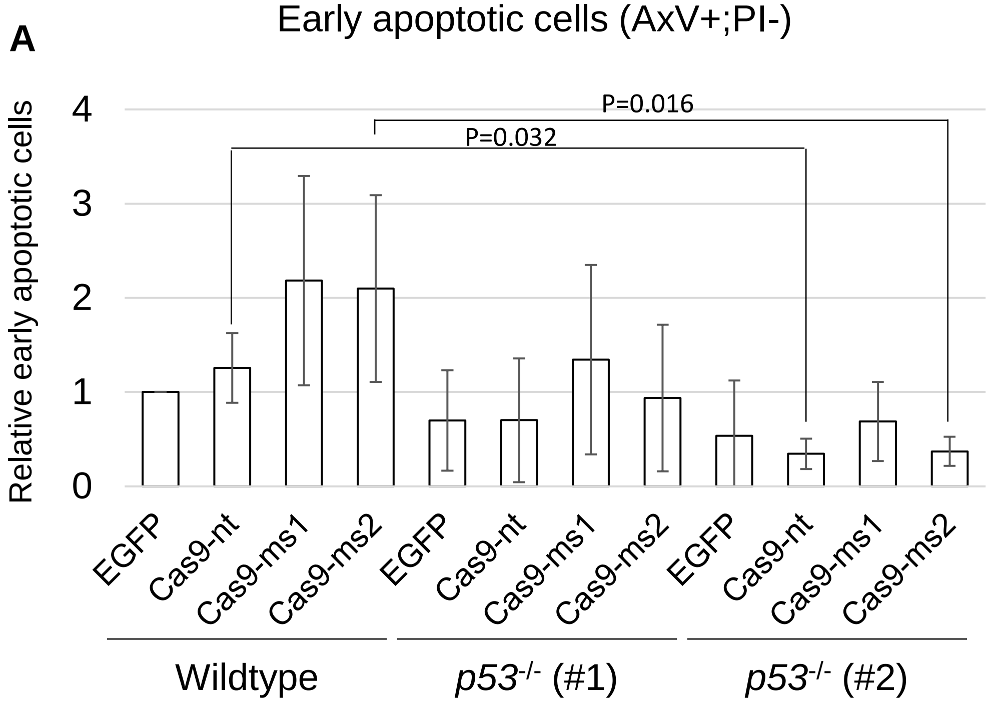

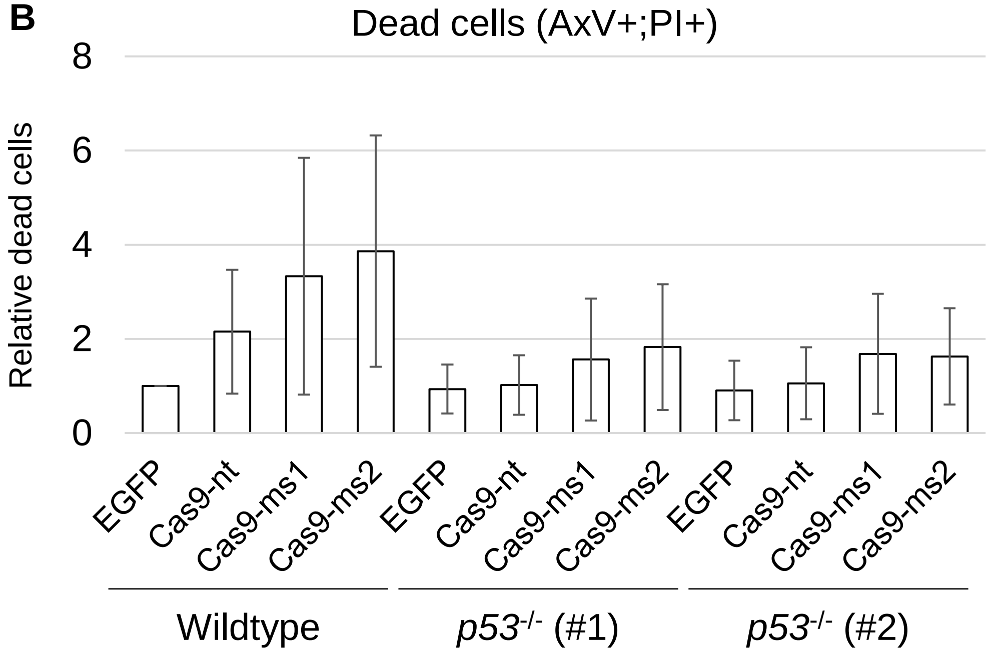

Figure S6. Involvement of p53 in mESCs functioning with the Cas9-sgRNA^{ms} system. Relative populations of early apoptotic cells (A) and dead cells (B) 72 h after transfection with Cas9-sgRNA^{ms} expression vectors in wildtype ESCs as control and two lines of *p53* knock-out mouse ESCs. Mean ± SEM. Two-way ANOVA was performed for the analysis of statistical differences. Significant differences were observed between the factor of ESC lines (P=8.4×10-5) in A, and both factors of vectors (P=0.016) and ESC lines (P=0.037) in B. Dunnett's test were performed after One-way ANOVA analysis for between-group comparisons. No significant differences were found in B. The experiment was conducted four times.

Figure S7

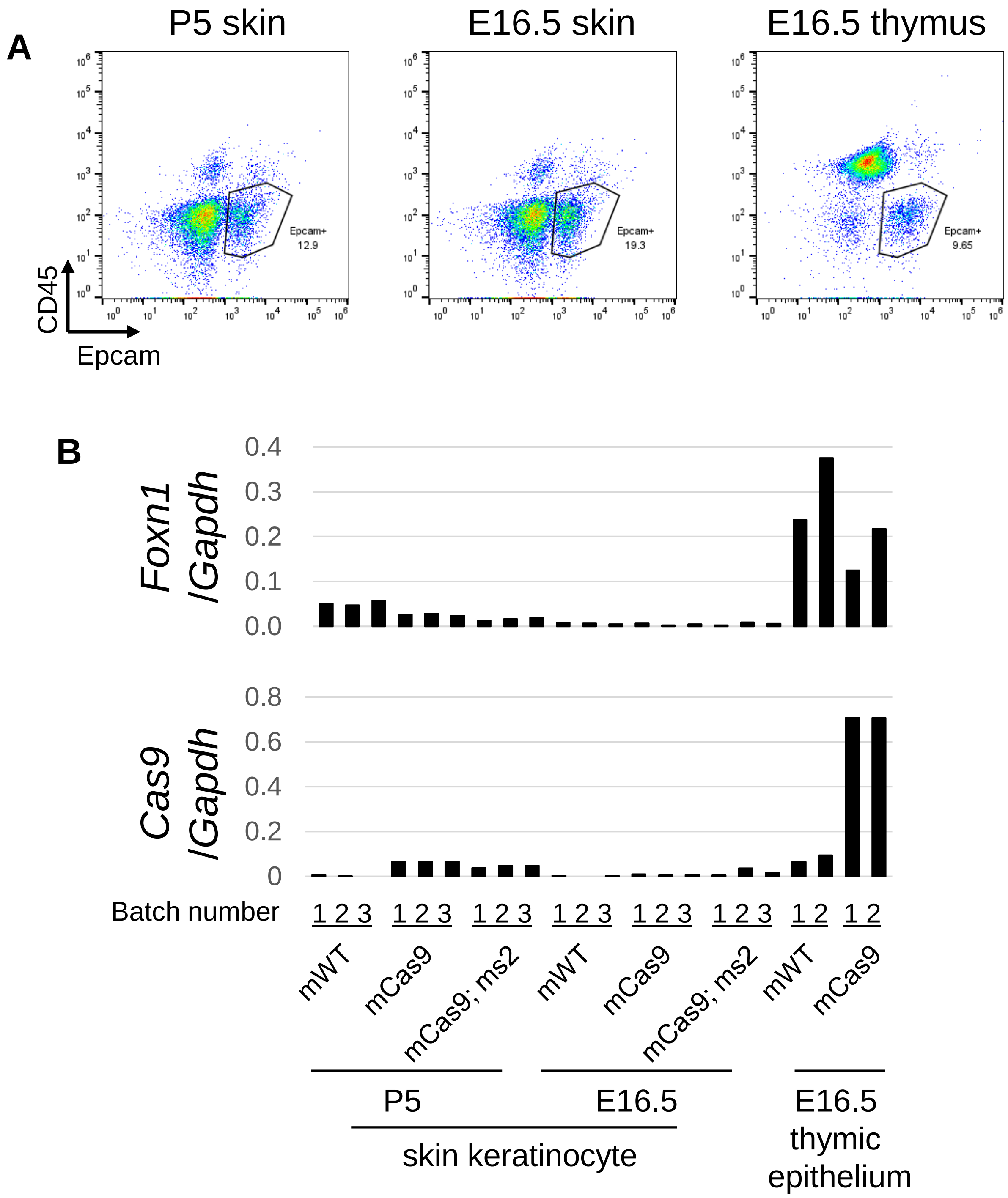

Figure S7. Foxn1 and Cas9 expression levels in the skin keratinocyte and the thymic epithelium
(A) Epcam-positive and CD45-negative cells were collected in each sample by FACS.
(B) cDNAs were prepared from Epcam-positive cells of 5 days after birth (P5) and embryonic day 16 (E16.5) skin, and embryonic day 16 (E16.5) thymus in the wild type mice (mWT), the Foxn1^{Cas9} mice (mCas9). Since the Foxn1^{Cas9}; R26_ms2 mice (mCas9: ms2) have absent thymus, the skin samples were analyzed only.

Figure S8

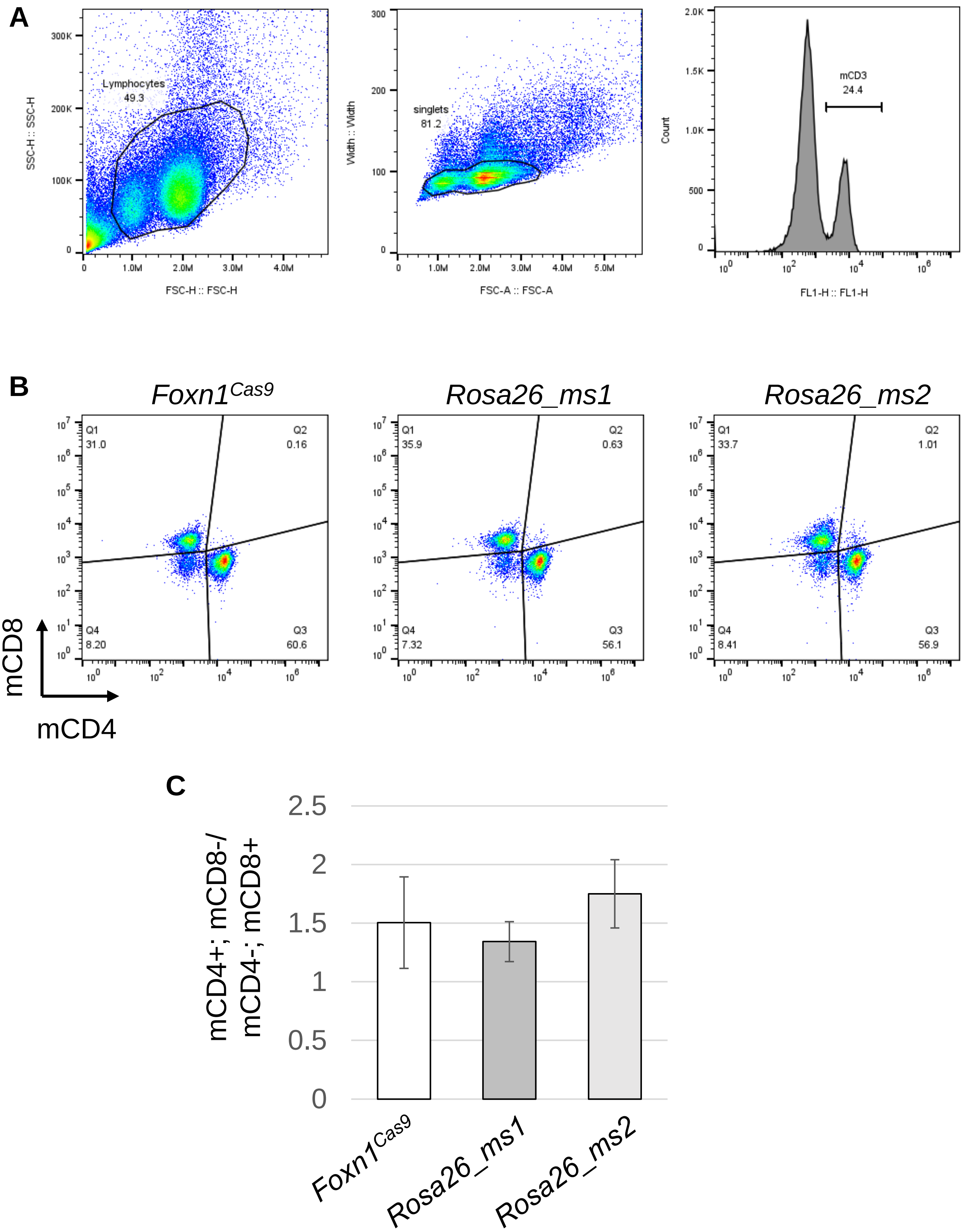

Figure S8. Peripheral T-cell population in Foxn1^{Cas9}, R26_ms mice lines. (A) Gates of moues CD3-positive population in the Foxn1^{Cas9} splenocytes for this assay. All splenocyte samples were gated the same. (B) The mouse CD4-positive and the mouse CD8-positive population in the mouse CD3-positive. (C) The ratio of mouse CD4 single positive and mouse CD8 single positive. This experiment was conducted by using four or more mice.

Lim JJY et al.,

Figure S9

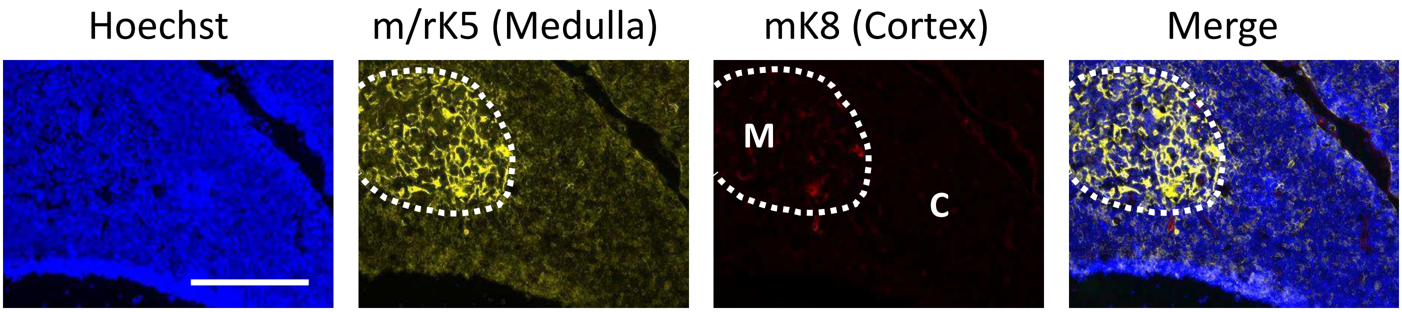

Figure S9. Immunohistochemistry of the 7-day-old rat thymus.
The anti-K5 antibody was detected in rat medullary thymic epithelial cells, whereas the anti-K8 antibody did not recognize rat cortical thymic epithelial cells, even in 7-day-old rats. Bar shows 200 µm.

Lim JJY et al.,

Figure S10

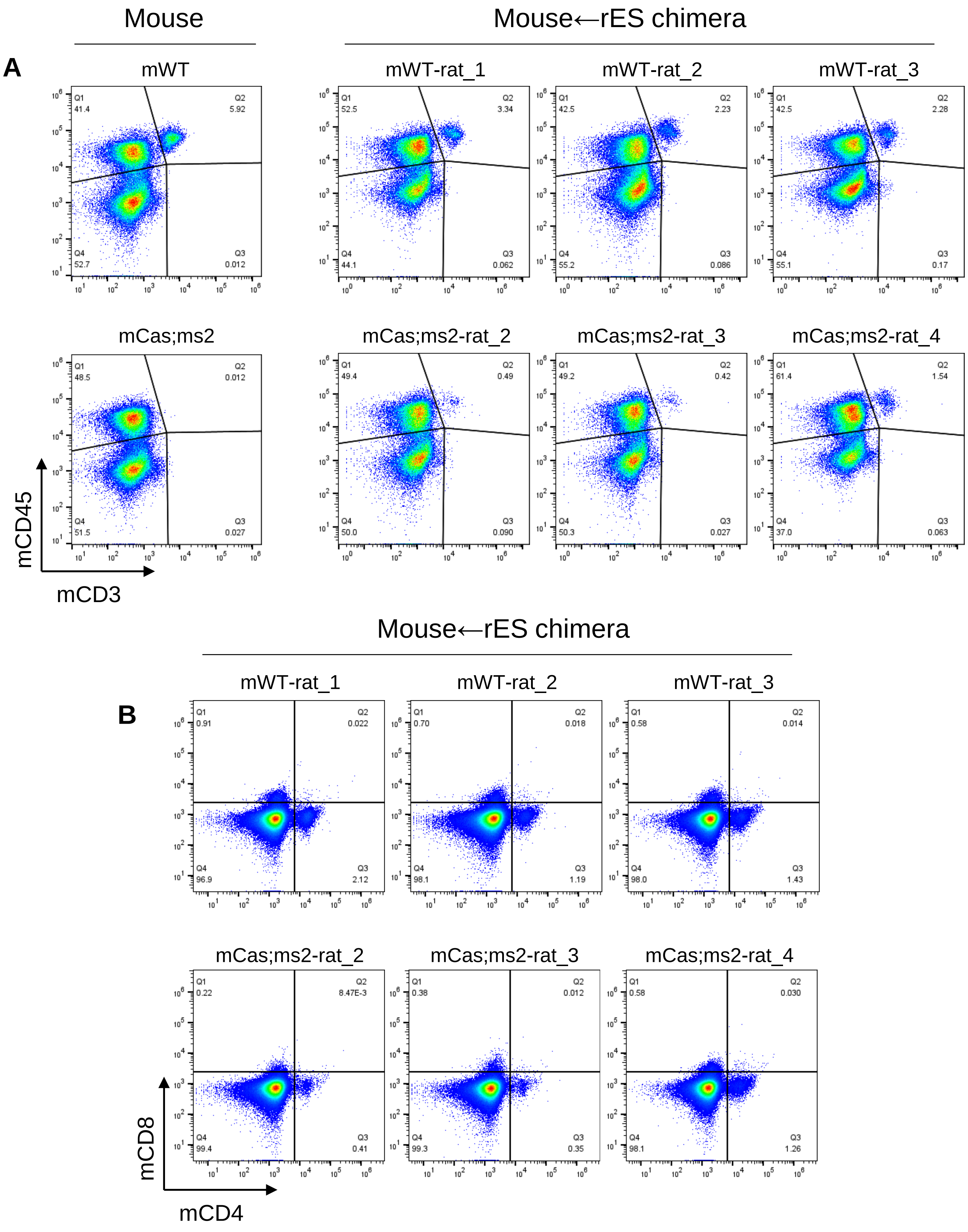

Figure S10 FCM analysis of splenocytes in the interspecies chimeras for mouse lymphocytes. (A) Analysis of mouse CD45 and mouse CD3. (B) Analysis of mouse CD4 and mouse CD8.

Lim JJY et al.,

Figure S11

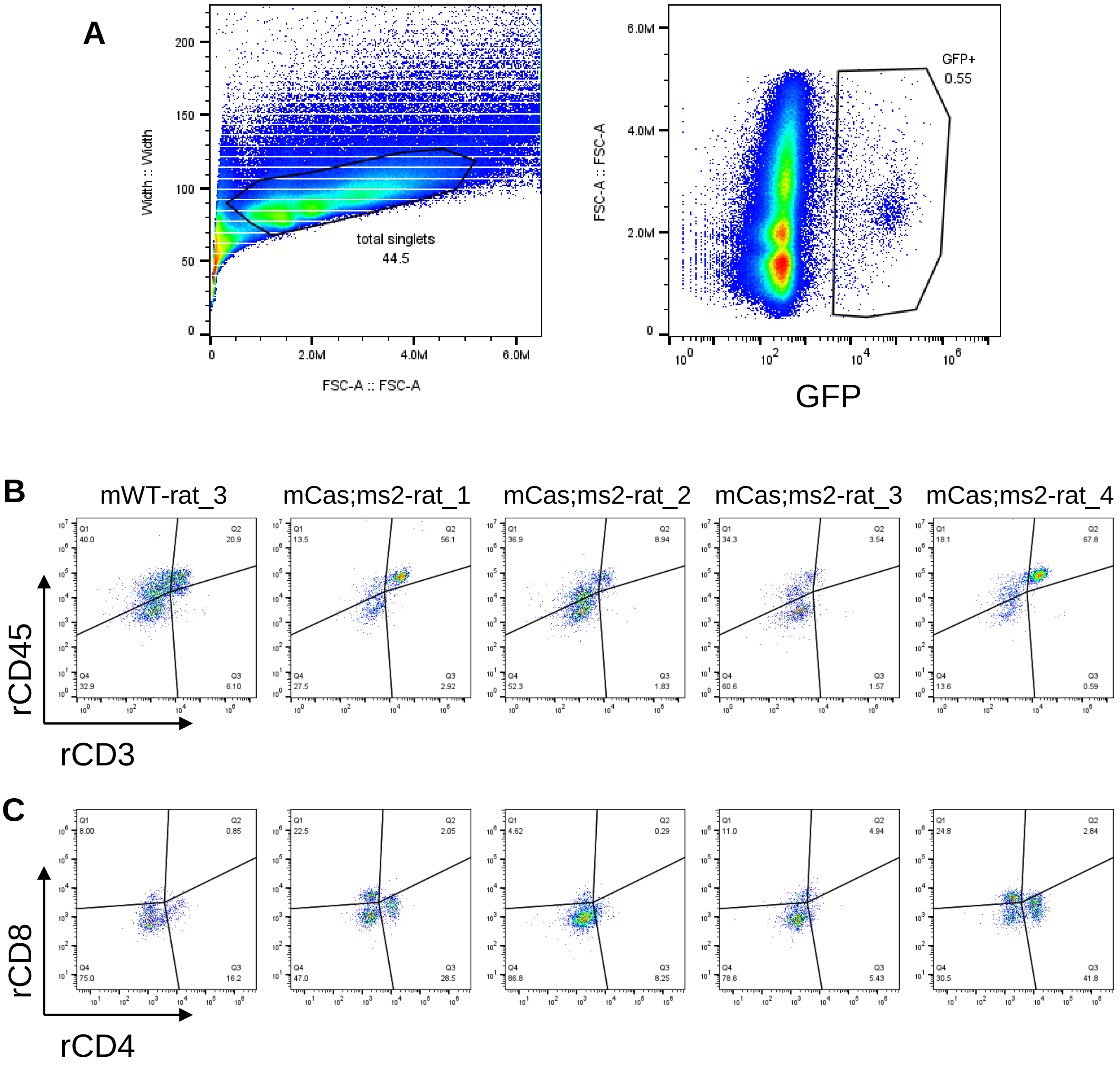

Figure S11 FCM analysis of splenocytes in the interspecies chimeras for rat lymphocytes.
(A) Gates of EGFP-positive population in the splenocytes of the interspecies chimeras for this assay. (B) Analysis of rat CD45 and rat CD3. (C) Analysis of rat CD4 and rat CD8.

Figure S12

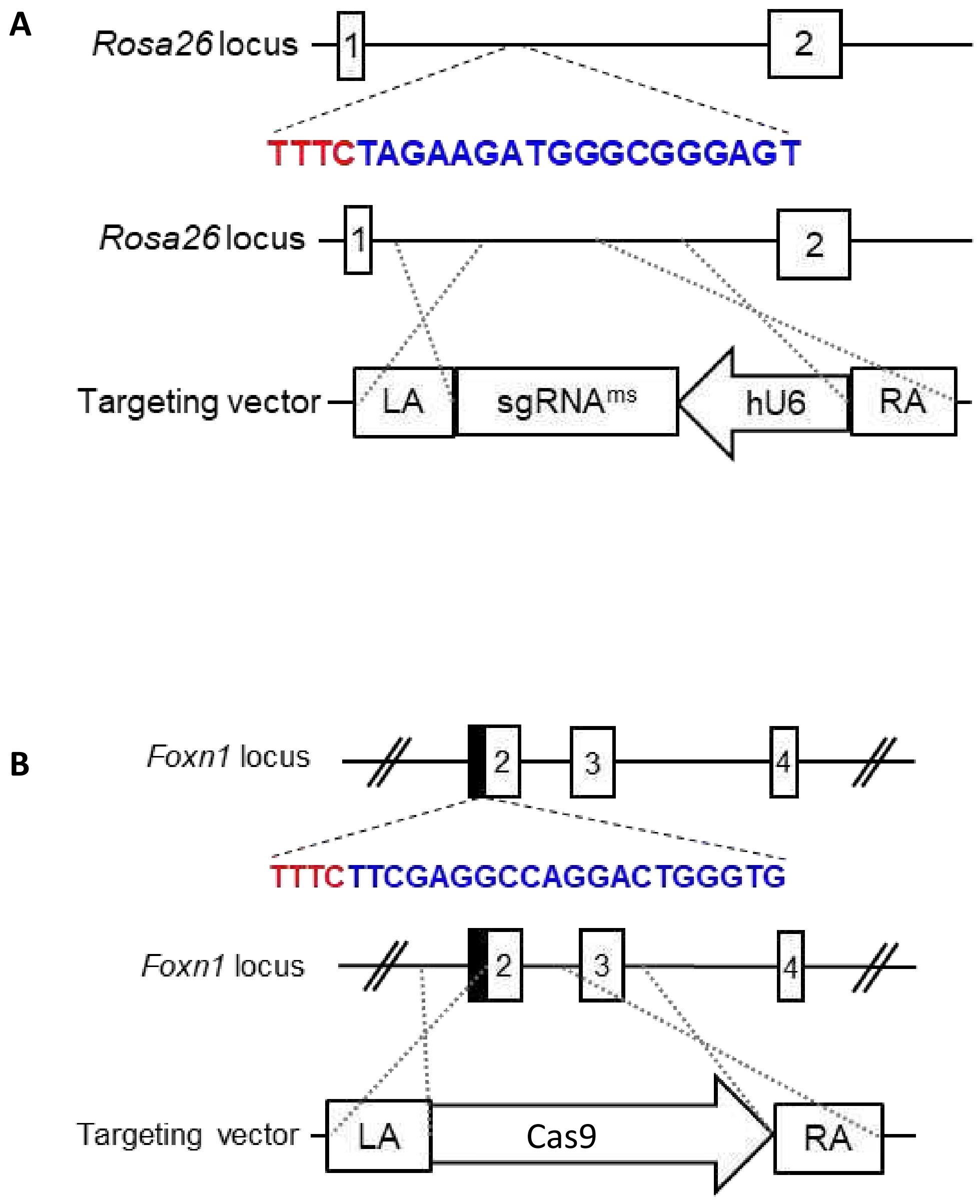

Figure S7. Schematic diagram of knock-in strategy at different loci.
(A) *Rosa26_ms*; (B) *Foxn1*^{Cas9} Red: PAM sequence of *CpfI*; Blue: crRNA sequence; LA: Left homologous arm; RA: Right homologous arm.

Supplementary table S1

Primer sets for construction of the targeting vectors

PCR product	Primer name	Sequence (5' – 3')
Foxn1 left arm	Foxn1-Left-FW	TGCCTGCAGGCTCTTCGATCTGTGTGCTTGTGCTGTGCTC
	FoxN1-Left-RV (SNP)	TCCTGGCCTCGAAGATAGCC
Foxn1 right arm	FoxN1-Right-FW (SNP)	GCTATCTTCGAGGCCAGGAACGCGTTAGACCCAGAAGGGCCAAGT
	Foxn1-Right-RV	TGGCAATGCCCGGGATGATTGCATAGGGGTGTGTTGGTC
Rosa26 left arm	Rosa26-left_F	ATGCCTGCAGGCTCTTCGATGAGAAGGGAGCGGAAAAGTC
	MluI+Rosa26-left-R	ACGCGTGACTGGAGTTGCAGATCACGAGGG
Rosa26 right arm	MluI+Rosa26-Right-F	TGCAACTCCAGTCACGCGTGGAATTGAACAGGTGTAAAATTGG
	Rosa26-Right_R	TGGCAATGCCCGGGATGATAATGCCATGAGTCAAGCCAG

Supplementary table S2

gRNA target sites

target gene	type of CRISPR	target sequence with PAM (5' – 3')
p53 (-5')	CRISPR-Cas9	ATTTCAGGAAACTTATGCGAGGG
p53 (-3')	CRISPR-Cas9	CTGGCTGGATAGAATTTCGCTGG
Rosa26	CRISPR-Cpf1	TTTCTAGAAGATGGGCGGGAGTCT
Foxn1	CRISPR-Cpf1	TTTCTTCGAGGCCAGGACTGGGTG

Under bars indicate PAM sequence.

Supplementary table S3

Primer sets for genotyping

Locus		Sequence (5' – 3')
p53 (for KO allele)	Fw	GGGTTTGAAGAATGGAGCTG
	Rv	TGCAGCCCACAGACTGA
p53 (for WT allele)	Fw	CCATGGCCATCTACAAGAAGT
	Rv	AACACGAACCTCAAAGCTGTC
Rosa26_ms	Fw	AGCTGCAGTGGAGTAGGCGG
	Rv	TGGAAAATACTCCGAGGCGG
Foxn1 (for WT allele)	Fw	GGTTACCCTCTGTGTCATTG
	Rv	GGAGTTTATTGCACCAAGCC
Foxn1^{Cas9}	Fw	CCAAAACTGAGCACAAAGGT
	Rv	AACAGGTCGGCGTACTGGTC

Supplementary table S4

Primer sets for qPCR

gene	Primer name	Sequence (5' – 3')
Gapdh	RT-mGapdh F	CATTTGCAGTGGCAAAGTGGAG
	RT-mGapdh R	CGTCAGATCCACGACGGAC
Foxn1	mFoxn1 F3	CGCAAAAGCATGGCCAAACC
	mFoxn1 R3	GTAGGTCCTGCAGGGGGTTC
Cas9	Cas9 seq F5	GAACCGCCCTGATCAAAAAG
	Cas9 seq R4	GCCCTTATCCCACACGATCT
